# Intercostal Lung Hernias Presenting After Minimally Invasive Cardiac Surgery

**DOI:** 10.21470/1678-9741-2023-0403

**Published:** 2024-09-03

**Authors:** Eric E. Vinck, Ricardo A. Zapata, Cristian A. Tarazona, Camilo Montoya Medina, Ubaldo E. Rivas, Juan C. Rendón, José J. Escobar, Omar A. Matar, Laura A. Gómez, Dora E. Lopera

**Affiliations:** 1 Department of Thoracic and Cardiovascular Surgery, Cardio VID Clinic, Pontifical Bolivarian University, Medellín, Antioquia, Colombia

**Keywords:** Hernia, Thoracotomy, Sternotomy, Mitral Valve, Video-Assisted Thoracic Surgery, Surgical Mesh, Cardiac Surgical Procedures, Lung

## Abstract

**Introduction:**

With the introduction of minimally invasive cardiac surgery, more commonly cases of lung herniation are starting to appear. Acquired lung hernias are classified as postoperative, traumatic, pathologic, and spontaneous. Up to 83% of lung hernias are intercostal. Herein, we describe patients presenting with intercostal lung hernias following minimally invasive cardiac surgery at a single center in Medellín, Colombia.

**Methods:**

We conducted a retrospective search of all patients presenting with intercostal lung hernias secondary to minimally invasive cardiac surgery at our clinic in Medellín since the beginning of our program, from 2010 to 2022. Mini-sternotomies were excluded from our study. We reviewed the incision type and other possible factors leading to intercostal lung hernia development. We also describe the approach taken for these patients.

**Results:**

From 2010 up until 2022, 803 adult patients underwent minimally invasive cardiac surgeries through a mini-thoracotomy. At the time of data retrieval, nine patients presented with intercostal lung hernias at the previous incision site. Five hernias (55%) were from right 2^nd^ intercostal parasternal mini-thoracotomies for aortic valve surgeries. Four hernias (45%) were from right 4^th^ intercostal lateral mini-thoracotomies for mitral valve surgeries. Our preferred repair technique is a video-assisted thoracoscopic mesh approach.

**Conclusion:**

Minimally invasive cardiac surgical approaches are becoming more routine. Proper wound closure is critical in preventing lung hernias. Additionally, timely diagnosis and opportune hernia surgery using video-assisted thoracoscopic mesh repair can prevent further complications.

## INTRODUCTION

In 1499, Roland was the first to describe lung herniation^[1]^. Morel-Lavalee further classified lung hernias according to their anatomical locations and whether they are acquired or congenital^[1]^. Acquired lung hernias are again classified as postoperative, traumatic, pathologic, and spontaneous^[1]^. Up to 83% of lung hernias are intercostal (IC)^[1]^. With the introduction of minimally invasive cardiac surgery (MICS), more and more cases of lung herniation are starting to appear^[1,2]^. Although the majority are right-sided hernias because of the right-sided approach to mitral valve repairs and aortic valve replacements (AVR), left-sided lung hernias may also appear secondary to minimally invasive direct coronary artery bypass (MIDCAB)^[2,3]^. Since the introduction of MICS in Colombia in 2010, our clinic in Medellín has been the epicenter for MICS in the country and the only Colombian center with over 300 MICS cases^[4,5]^. During this time, a total of 803 adult patients underwent MICS through mini-thoracotomies. Of these, seven were left mini-thoracotomies for MIDCABs. At the time of data collection (12 years of MICS), nine patients developed IC lung hernias secondary to MICS; an incidence of 0.01%. To date, no lung hernias secondary to robotic cardiac surgeries have been reported in Colombia (one center in Bogota performs robotic cardiac surgery)^[6]^. Of the nine patients who developed lung hernias, eight were taken to surgical correction while one asymptomatic patient is in routine follow-up.

## METHODS

We conducted a retrospective search of all patients presenting with IC lung hernias secondary to MICS at our clinic in Medellín, Colombia, since the beginning of our program, from 2010 to 2022. Mini-sternotomies were excluded from our study. We reviewed MICS incision type and other possible factors leading to IC lung hernia development. We also describe the approach taken for these patients. Ethics board approval was obtained and patient consent was given.

## RESULTS

**Fig. 1:**
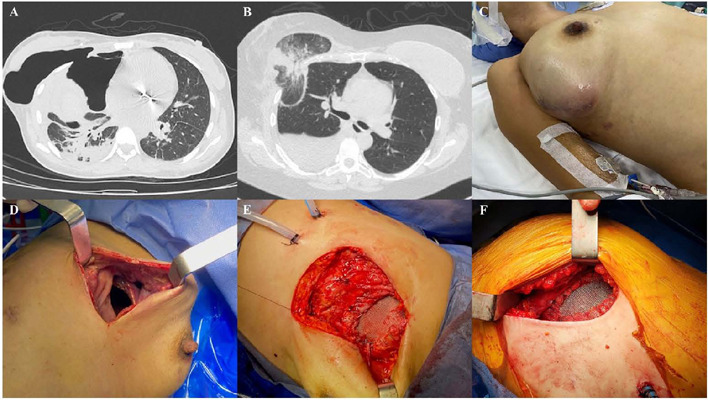
A-B) Chest computed tomography showing a right-sided intercostal defect with a pneumothorax and protrusion of lung parenchyma into the herniated space. C) Preoperative image showing a bulging mass into the right chest of the patient. D) Right intercostal defect after lung reduction revealing wide intercostal space. E) Direct open mesh hernia repair. F) Post-video-assisted thoracoscope mesh lung hernia repair.

Since the start of our MICS program in 2010 up until 2022, 803 adult patients underwent MICS through a mini-thoracotomy. Of these, seven had left mini-thoracotomies for a MIDCAB approach (Table 1). MICS surgeries included mitral valve repair or replacement (MVR), AVR, and atrial septal defect closures. At the time of data retrieval, nine patients presented with IC lung hernias at the previous incision site. Five patients (55%) were female, and four patients (45%) were male. Five hernias (55%) were from right 2^nd^ IC parasternal minithoracotomies for AVRs. Four hernias (45%) were from right 4^th^ IC lateral mini-thoracotomies for MVRs. Two hernias (one AVR) and (one MVR) developed following postoperative reintervention through the same MICS incision to control bleeding (Table 1). Average time from the first MICS surgery to lung hernia development was 1.5 months, while average time from hernia diagnosis to hernia correction was four months. One exception was a patient who had her hernia corrected three years after diagnosis. Patients present initially with IC pain with a bulging mass in the hernia site and intermittent dyspnea. Chest computed tomography scans reveal the herniated lung and pleural space (Figure 1A-B). The surgical approach used for lung hernia repair in these patients involves hernia reduction, hernia sac resection, adhesion lysis, decortication depending on intraoperative findings, and mesh repair through a video-assisted thoracoscopic (VATS) technique (Figure 1C-F). We use a polypropylene mesh and polydioxanone sutures for rib approximation and closure of the augmented IC space. Six patients had a VATS approach without using a Finochietto rib spreader. One patient required both rib spreading and VATS, while another patient had a direct open thoracic wall reconstruction without VATS nor rib spreading.

**Table 1:** Characteristics of patients with lung hernias following MICS in Medellín, Colombia.

Patient no.	Sex	Age (years)	Primary surgery	Time of hernia	Same incision re-intervention	Hernia characteristics	Comorbidities	Surgery technique
1	Female	51	AVR	3 years	None	2^nd^ IC, right parasternal	Takayasu arteritis, AHT	VATS, mesh repair
2	Male	65	AVR	2 months	Postoperative bleeding	2^nd^ IC, right parasternal	AHT, epilepsy	VATS, mesh repair
3	Male	77	MVR	1 month	None	4^th^ IC, right lateral	Abdominal aortic aneurysm, peripheral arterial disease	VATS, mesh repair
4	Female	63	AVR	2 months	None	2^nd^ IC, right parasternal	AHT, obesity, prediabetes, hiatal hernia, fatty liver	VATS, mesh repair
5	Female	53	Mitral annuloplasty	1 month	None	4^th^ IC, right lateral, periareolar	AHT, obesity, hypothyroidism	VATS, mesh repair
6	Female	32	MVR	1 month	Postoperative bleeding	4^th^ IC, right lateral	Hydrocephaly (pediatric)	Open mesh repair
7	Male	71	MVR + maze + tricuspid valve repair	1 month	None	4^th^ IC, right lateral	AHT, dyslipidemia, atrial fibrillation	VATS, mesh repair
8	Male	70	AVR	1 month	None	2^nd^ IC, right parasternal	AHT, dyslipidemia	VATS, mesh repair

AHT=arterial hypertension; AVR=aortic valve replacement; IC=intercostal; MICS=minimally invasive cardiac surgery; MVR=mitral valve repair or replacement; VATS=video-assisted thoracoscopic surgery

## DISCUSSION

Up until 2002, only three cases of lung hernias following MICS were reported^171^. Although the exact incidence of lung hernias is unknown, some centers are beginning to report cases following MICS and soon incidence reports will start to surface. Table 2 outlines recent reports of IC lung hernias following MICS. Although the exact cause of lung hernia development is not known, improper chest wall closure and severe coughing seem to be important contributing factors. In 2020, Cetinkaya et al. reported 20 cases of lung hernias at a German center from a subset of 1,381 patients indicating an incidence of 0.01% during seven years^[8]^. This number agrees with the incidence reported here by our center also at 0.01%. In 2009, Santini et al. described a VATS approach for lung hernia repair followed by Cafarotti in 2014^[9,10]^. At our clinic, VATS is the technique and approach of choice. Robot-assisted cardiac surgery is also subject to the development of IC lung hernias^[1,11]^. Although symptomatic lung hernias require surgical repair, in some cases manual repositioning may be an option^[12]^. In fact, smaller asymptomatic hernias may not require surgery, and these patients can be followed on an outpatient basis keeping in mind the risk of lung strangulation and/or symptom development. Because of the rare entity and low incidence of IC lung hernia development especially following MICS, true indications of surgery are still not standardized. As for large IC defects and symptomatic patients, surgery should be considered^[13,14,15]^. The best treatment approach remains hernia prevention, therefore, wound closure should be meticulous and carefully performed ensuring proper rib approximation.

**Table 2:** Latest published cases of lung hernias following MICS.

Author	Year	Patient’s sex	Patient’s age (years)	Primary surgery	Time of hernia appearance	Hernia characteristic
Deeik	1998	Male	66	MIDCAB	1 month	4^th^ IC, left
Gouda	2002	Male	36	MICS, mitral	6 weeks	Not stated
Athanassiadi	2007	Male (12)	Between 23-77	Not stated	Not stated	Right (8)
		Female (4)				Left (6) Bilateral (2)
Santini	2008	Female	59	MICS, mitral	7 months	4^th^ IC, right
Wiedemann	2011	Not stated	50	ASD repair	Not stated	4^th^ IC, right
Waymann	2011	Male	67	MIDCAB	1 year	3^rd^ IC, left
Bhamidipati	2012	Male	60	Robotic mitral annuloplasty	1 year	3^rd^ IC, right
		Male	48	Robotic mitral annuloplasty	1.5 years	4^th^ IC, right
Cafarotti	2013	Not stated	Not stated	MICS, aortic valve	5 years	Not stated
Kumar	2013	Female	62	VATS, pulmonary vein ablation	6 weeks	7^th^ IC, left
Chen	2014	Male	29	MICS, mitral	5 years	4^th^ IC, right
Wilgus	2018	Male	52	Robotic mitral valve repair	4 months	4^th^ IC, right
Meana	2018	Male	87	MICS, mitral repair	10 years	Not stated
Koichi	2019	Female	51	MICS, mitral annuloplasty	5 days	4^th^ IC, right

ASD=atrial septal defect; IC=intercostal; MICS=minimally invasive cardiac surgery; MIDCAB=minimally invasive direct coronary artery bypass; VATS=video-assisted thoracoscopic surgery

## CONCLUSION

Minimally invasive cardiac surgical approaches are becoming more routine. This progressive increase in smaller incisions also introduces newer challenges and possible complications which demand more from the surgeon. Proper wound closure is critical in preventing lung hernias. Additionally, timely diagnosis and opportune hernia surgery using VATS mesh repair can prevent further complications.

## Abbreviations, Acronyms & Symbols

AHT: Arterial hypertension
ASD: Atrial septal defect
AVR: Aortic valve replacements
IC: Intercostal
MICS: Minimally invasive cardiac surgery
MIDCAB: Minimally invasive direct coronary artery bypass
MVR: Mitral valve repair or replacement
VATS: Video-assisted thoracoscopic surgery
